# Association between specific dystrophin gene mutations and myocardial fibrosis by cardiovascular magnetic resonance imaging in patients with Duchenne and Becker muscular dystrophy

**DOI:** 10.1186/1532-429X-16-S1-P325

**Published:** 2014-01-16

**Authors:** Marly C Silva, Carlos H Rassi, Zilda M Meira, Juliana G Giannetti, Mariz Vainzof, Mayana Zatz, Roberto Kalil, Carlos E Rochitte

**Affiliations:** 1 Cardiology, Heart Institute, InCor, University of São Paulo Medical School, São Paulo, São Paulo, Brazil; 2 Cardiology and Pediatry, Federal University of Minas Gerais, Belo Horizonte, Minas Gerais, Brazil; 3 Human Genome Research Center, Bioscience Institute, University of São Paulo, São Paulo, São Paulo, Brazil; 4 Radiology, Axial Centro de Imagem, Belo Horizonte, Minas Gerais, Brazil

## Background

Duchenne (DMD) and Becker (BMD) muscular dystrophies (MD) are allelic X-linked recessive disorders, caused by mutation of the dystrophin gene located at locus Xp21 that consists of 79 exons, characterized by progressive skeletal muscle degeneration and replacement by fibro fatty tissue. Dystrophin is a sarcolemal protein that links the cytoskeleton to the basal lamina and is essential for maintenance of the muscular membrane integrity during muscular contraction. Cardiac involvement is frequent, 70 - 80% of patients, and often develops clinically silent, without any evident early clinical signs. CMR can identify myocardial fibrosis (MF) and may be useful for detecting the early stages of cardiomyopathy in MD. In a previous study, DNA analyses in 47 pts with DMD revealed significant association between dilated cardiomyopathy (DCM) and specific exons and possible protection against DCM by other exons. The association between specific exons mutation of the dystrophin gene and myocardial fibrosis is until unknown.

## Methods

We enrolled 76 pts, 70 pts DMD and 6 BMD with confirmed muscular dystrophy. Mean age was 13.1 ± 4.4 years. MLPA tests were performed on all these patients. 40 pts showed DNA mutation. According to specific exons mutation, these patients were classified into 2 groups: with exons mutation below 45 and with mutation on or above 45. All patients underwent CMR study in a 1.5-T Siemens Avanto (Erlangen, Germany), using cine resonance for function evaluation and myocardial delayed enhancement (MDE) technique for myocardial fibrosis detection, 10 min after intravenous bolus of 0.2 mmol/kg of gadolinium-based contrast. Cine and MDE parameters were, respectively: TR 2.0/9.0, TE 1.07/5.0; FA 69/50; cardiac phases 20/1; VPS 8/16 to 32; matrix 192 × 162/256 × 192; ST 8/8 mm; gap 2/2 and FOV 32-38/32-38 cm; TI none/150-390 ms. Two experienced observers measured LV volumes and ejection fraction by the Simpson method (Argus software, Siemens). In the MDE short axis images, we evaluated the MF mass per patient, using a 5 standard deviation thresholding technique, on CMR42 software, version 3.4.2 (Circle Cardiovascular Imaging, Calgary, Alberta, Canada). Mann Whitney was used for comparison of myocardial fibrosis between the group with mutation in exons < 45 and mutation in exons ≥45.

## Results

Of the 40 pts with DNA mutation, 16 pts showed mutation below exon 45 and 25 pts in exons on or above 45. The specific mutations were shown to be associated with cardiac involvement (myocardial fibrosis). Patients with mutations in exons less than 45 had greater extent of myocardial fibrosis than patients with mutations in exons greater than or equal to 45 in CMR at baseline (27.9 ± 18.4% vs. 12.1 ± 13.4%, respectively, p = 0.006) and at follow-up (33.1 ± 21.1% vs. 18.8 ± 16.9%, respectively, p = 0.024).

## Conclusions

There was a significant correlation between the site of mutation in the dystrophin gene and myocardial fibrosis. Mutations in exon ≥ 45 appear to protect against cardiac involvement.

## Funding

No funding.

**Figure 1:**
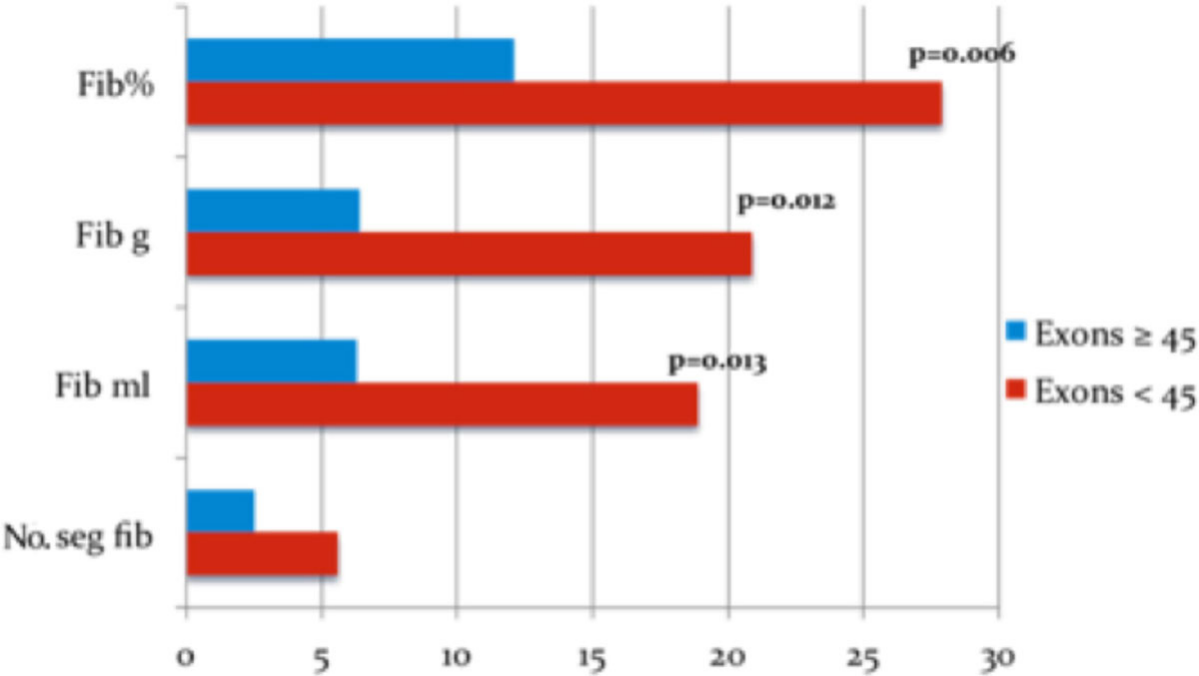
**Myocardial fibrosis quantification in DMD and BMD patients with mutation in exons ≥ 45 and < 45**. Fib% - myocardial fibrosis as percent of LV mass Fib g - myocardial fibrosis in grams Fib ml - myocardial fibrosis volume in milliliters No. seg. fib - number of AHA segments with fibrosis.

